# Myocardial Segmentation of Cardiac MRI Sequences With Temporal Consistency for Coronary Artery Disease Diagnosis

**DOI:** 10.3389/fcvm.2022.804442

**Published:** 2022-02-25

**Authors:** Yutian Chen, Wen Xie, Jiawei Zhang, Hailong Qiu, Dewen Zeng, Yiyu Shi, Haiyun Yuan, Jian Zhuang, Qianjun Jia, Yanchun Zhang, Yuhao Dong, Meiping Huang, Xiaowei Xu

**Affiliations:** ^1^Guangdong Provincial People's Hospital, Guangdong Academy of Medical Sciences, Guangzhou, China; ^2^Guangdong Provincial Key Laboratory of South China Structural Heart Disease, Guangdong Cardiovascular Institute, Guangzhou, China; ^3^Department of Computer Science, Carnegie Mellon University, Pittsburgh, PA, United States; ^4^Shanghai Key Laboratory of Data Science, School of Computer Science, Fudan University, Shanghai, China; ^5^Cyberspace Institute of Advanced Technology, Guangzhou University, Guangzhou, China; ^6^Department of Computer Science and Engineering, University of Notre Dame, Notre Dame, IN, United States; ^7^Department of Catheterization Lab, Guangdong Provincial People's Hospital, Guangdong Academy of Medical Sciences, Guangzhou, China

**Keywords:** myocardial segmentation, MRI, cardiac sequences, temporal consistency, coronary artery disease, diagnosis

## Abstract

Coronary artery disease (CAD) is the most common cause of death globally, and its diagnosis is usually based on manual myocardial (MYO) segmentation of MRI sequences. As manual segmentation is tedious, time-consuming, and with low replicability, automatic MYO segmentation using machine learning techniques has been widely explored recently. However, almost all the existing methods treat the input MRI sequences independently, which fails to capture the temporal information between sequences, e.g., the shape and location information of the myocardium in sequences along time. In this article, we propose a MYO segmentation framework for sequence of cardiac MRI (CMR) scanning images of the left ventricular (LV) cavity, right ventricular (RV) cavity, and myocardium. Specifically, we propose to combine conventional neural networks and recurrent neural networks to incorporate temporal information between sequences to ensure temporal consistency. We evaluated our framework on the automated cardiac diagnosis challenge (ACDC) dataset. The experiment results demonstrate that our framework can improve the segmentation accuracy by up to 2% in the Dice coefficient.

## 1. Introduction

Coronary artery disease (CAD) is the most common cause of death globally. It affects more than 100 million people, and results in about 10 million death each year (1). In the United States, about 20% of those over 65 have CAD (2). MRI is a common tool for CAD diagnosis. With cardiac magnetic resonance (CMR), the myocardial structure and functionality can be assessed and analyzed. Particularly, experienced radiologists manually perform MYO segmentation on the CMR image sequences and measure several parameters to finally determine the diagnosis. For instance, the left and right ventricular (RV) ejection fractions (EF) and stroke volumes (SV) are widely used for cardiac function analysis (3).

Recently, automatic MYO segmentation of CMR image sequences has attracted considerable attention in the community (4, 5). On the one hand, with the aging society, the number of patients with CAD has been increasing for decades (6). On the other hand, manual MYO segmentation is tedious, time-consuming, and with low replicability. Considering the medical cost and quality, automatic MYO segmentation is highly desirable. However, it is a challenging task. First, there exist large shape variations in the images. Second, the labels of the noisy images are with low uniformity, which degrades the training efficiency and effectiveness.

Currently, there exist two approaches for automatic MYO segmentation. In the traditional MYO segmentation approach (7, 8), a manually defined contour or boundary is needed for initialization. Although an automatic initialization might be achieved by some algorithms (9, 10), the segmentation performance highly relies on the initialization quality, which makes the framework lack stability. Another approach (11, 12) uses deep learning for MYO segmentation, which does not need any initialization and the whole process can run without manual inter-action. However, these methods treat each CMR frame independently, which does not exploit the temporal consistency among sequences.

On the other hand, temporal consistency has been extensively used to improve segmentation accuracy in a variety of applications. Yan et al. (13) added temporal consistency in the left ventricle segmentation from cine MRI. Punithakumar et al. (14) investigated automatic segmentation of the RV endocardial borders in 3D+ time magnetic resonance sequences acquired from patients with hypoplastic left heart syndrome (HLHS). Qin et al. (15) proposed a novel deep learning method for joint estimation of motion and segmentation from cardiac MR image sequences. Guo et al. (16) focused on automated segmentation of left ventricular (LV) in temporal cardiac image sequences. Recently, temporal consistency has been explored in other cardiac imaging modalities including MRI, CT, and ultrasound (US), which is detailed in Hernandez et al. (17). As far as we know, the effectiveness of temporal consistency has not been explored currently for MYO segmentation of CMR sequences.

In this article, we propose to exploit temporal consistency for MYO segmentation of CMR sequences for automatic CAD diagnosis. Particularly, we propose to combine conventional neural networks and recurrent neural networks to perform segmentation in two stages. The first stage contains an initial segmentation network (ISN) to get the initial segmentation without temporal information, while the second includes a temporal consistency based network (TCN) for refinement considering temporal consistency. The contributions of our study are:

- We proposed to use temporal consistency for accurate MYO segmentation of CMR sequence, and our framework is able to incorporate temporal information between CMR frames.
- To further exploit the temporal consistency among frames, we adopted a bi-directional training approach that can reduce segmentation error introduced by the first few frames in the training process.
- We conducted comprehensive experiments on the ACDC dataset. Compared with the residual 3D U-net model of Yang et al. (18), our framework achieves an improvement of 1–2% of segmentation accuracy in the Dice coefficient.

## 2. Background

### 2.1. MYO Segmentation of CMR Image

Cardiac MRI image is a widely used imaging tool for the assessment of MYO micro-circulation. It utilizes the electromagnetic signal with characteristic frequency produced by the hydrogen nuclei under a strong contrasting magnetic field and weak oscillating near field as the imaging agent.

Due to the high capacity for discriminating different types of tissues, CMR image is one of the most prominent standards for cardiac diagnosis through the assessment of the left and right ventricular EF and SV, the left ventricle mass and the myocardium thickness. For example, Bernard et al. (3) obtained these parameters from CMR images using an accurate segmentation of CMR image for the LV cavity, RV cavity, and the myocardium at end-diastolic (ED) frame and end-systolic (ES) frame can give out an accurate diagnostic of cardiac function.

**Figure 1:**
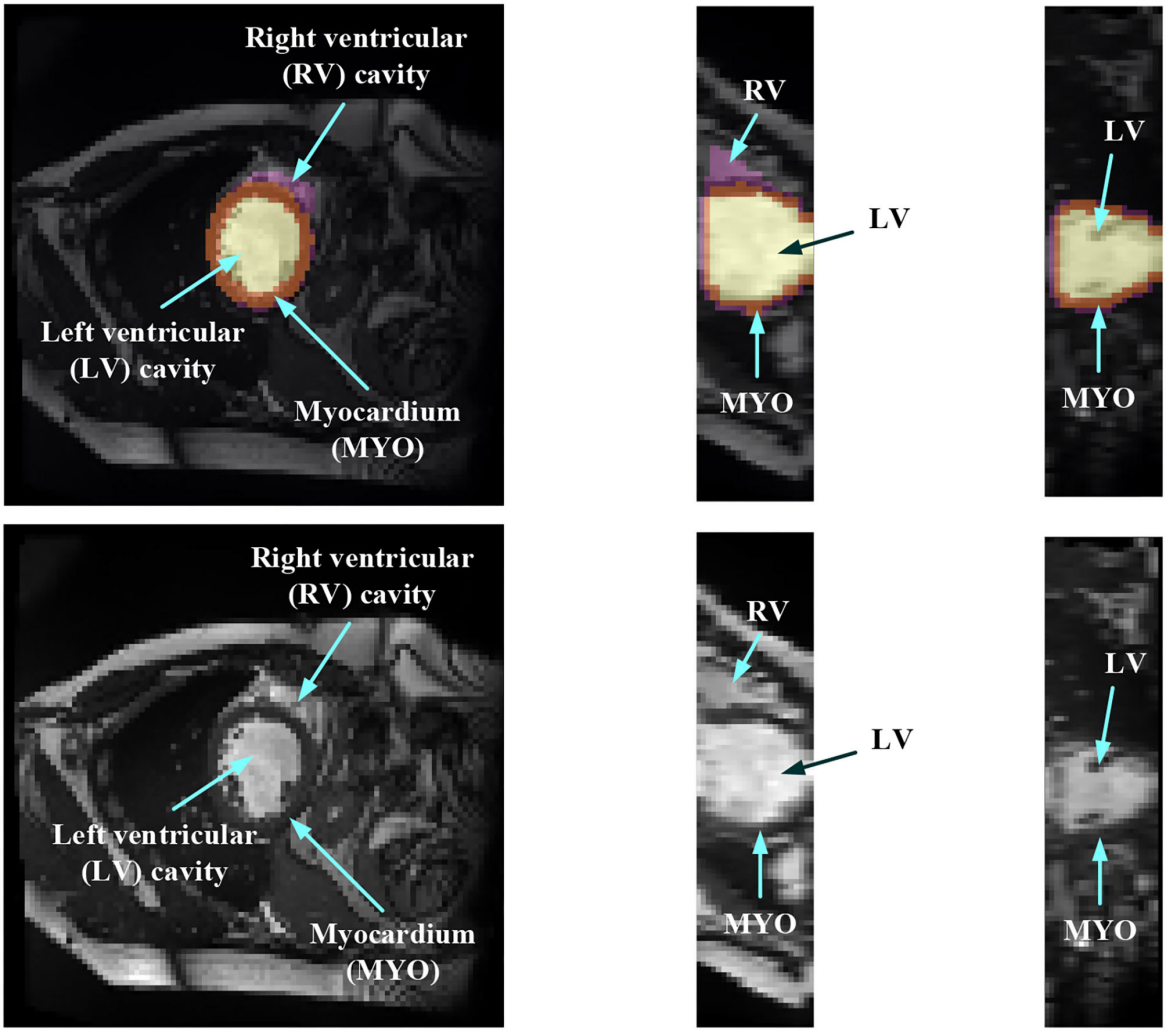
Structure illustration of a typical CMR image. The images are the slices on the *z*-axis, *y*-axis, and *x*-axis, respectively, from Patient 001 in the automated cardiac diagnosis challenge (ACDC) dataset at end-diastolic (ED) frame with mask, and the second row of the figure is the raw CMR slices of Patient 001.

In order to evaluate the MYO function, accurate segmentation of the LV cavity, RV cavity, and MYO need to be acquired from the framework. Figure 1 shows the slices of typical CMR images of a patient at ED frame with and without ground truth mask along each axis, respectively. The label shows the ground truth of segment results for different parts of the CMR image.

### 2.2. Related Study

Medical image segmentation has attracted much attention recently (19–26). MYO segmentation of CMR sequences has the following challenges. First, the contrast between the myocardium and surrounding structures is low as shown in Figure 1. Second, the brightness heterogeneity in the left and RV cavities is due to blood flow (3). Third, misleading structures such as papillary muscle have the same intensity and grayscale information as myocardium, which makes it hard to extract the accurate boundary. There are two approaches among existing works toward myocardium segmentation.

The first approach is based on point distribution models (PDMs) (27). A good example is the active shape model (ASM) (28) or active appearance model (AAM) (29). The main idea of ASM is to learn patterns of variability from a training set of correctly annotated images. ASM uses principal component analysis (PCA) to build a statistical shape model from a set of training shapes and then fits an image in a way that is most similar to the statistical shape in the training set. Ordas et al. (30) proposed an algorithm for MYO and LV cavity segmentation in CMR images based on invariant optimal feature 3-D ASM (IOF-ASM). van Assen et al. (31) improved the ASM such that the method can work for sparse and arbitrary oriented CMR images. Tobon-Gomez et al. (32) proposed a new ASM model that includes the measurement of reliability during the matching process to increase the robustness of the model. Santiago et al. (33) proposed a method of applying ASM on CMR images with the varying number of slices to perform segmentation on arbitrary slices of CMR images with a new re-sampling strategy.

The prediction results of ASM must be constrained into certain shape variations so that the shape of the segmentation result does not go too far from the regular myocardium shape. Note that this is very important when artifacts and defects in the CMR image make the myocardium boundary unclear and hard to recognize. However, ASM is based on linear intensity information in the image, which is insufficient to model the appearance of CMR data with huge intensity variations and large artifacts. In addition, ASM requires a manual initialization shape and the final segmentation result is very sensitive to the shape and position of this initialization. Thus, a fully automatic and non-linear model is needed.

The second approach adopts machine learning techniques to perform image segmentation. For example, Zhang et al. (34) used a simple implementation of a fully connected neural network for the quality assessment of CMR images. Poudel et al. (35) used a recurrent fully connect network (RFCN) on the stack of 2D images for the segmentation of CMR images. The recurrent network is applied on the short axis so that the continuous spatial information on the short axis can be utilized. Simantiris et al. (36) proposed to use Dilation CNN, where each layer has the same resolution so that the localized information in the input image would not be lost. Isensee et al. (37) proposed a multi-structure segmentation for each time step of MRI sequences and extracted the domain-specific features. Simantiris et al. (38) used a simple network composed of cascaded modules of dilated convolutions with increasing dilation rate without using concatenation or operations like pooling that will lead to the decrease of resolution. Zotti et al. (39) introduced the shape prior obtained from the training dataset in the 3D Grid-net and employed the contour loss as loss function to improve the performance on the border of segmentation result. Painchaud et al. (40) presented a method to guarantee the anatomical plausibility of segmentation results such that the anatomical invalid segmentation result of the model will be reduced to zero. Khened et al. (41) proposed a neural network with a dense block that contains dense connections between layers inspired by the Dense Net. Baumgartner et al. (42) compared the performance of 2D and 3D fully convolution network (FCN) and U-net. Calisto and Lai-Yuen (43) used a multi-objective evolutionary-based algorithm to incorporate 2D FCN and 3D FCN to search for an efficient and high-performing architecture automatically. Wolterink et al. (44) used six different types of model's average probability maps and cyclic learning rate schedule to improve the segmentation performance. Rohé et al. (45) proposed a combination of rigid alignment, non-rigid diffeomorphism registration, and label fusion to increase the performance of 3D U-net. Zotti et al. (46) used the shape prior that is embedded in the GridNet to reduce the anatomical impossible segmentation result. Patravali et al. (47) used the combination of 2D and 3D U-net and proposed a new class-balanced Dice loss to make the optimization easier.

Although these above methods showed great improvements in the segmentation performance compared to ASM or AAM. They treat each frame independently, which makes the segmentation results of some specific sequences inaccurate or the overall results lack coherence.

On the other hand, temporal consistency has been extensively used to improve the segmentation accuracy in various applications in multiple image modalities, which includes US, computerized tomography (CT) and CMR. Painchaud et al. (40) proposed a framework to deeply study the temporal consistency of 2D+ time segmentation methods in the US. Wei et al. (48) presented a co-learning model for temporal-consistent heart segmentation of echocardiographic sequences with sparsely labeled data. Guo et al. (16) leveraged to aggregate the characteristics of the spatial sequential network (SS-Net) to improve the LV segmentation during cardiac systole, and associated with excellent performance for temporal left ventricle segmentation on 4D cardiac CT sequence by sequential consistency (cardiac motion), achieving higher accuracy compared to the state-of-the-art methods on 4D cardiac CT dataset. Yan et al. (13) added temporal consistency in the left ventricle segmentation from cine MRI. Punithakumar et al. (14) investigated automatic segmentation of the RV endocardial borders in 3D+ time magnetic resonance sequences acquired from patients with HLHS. Zhang et al. (49) proposed a method to improve the segmentation of the left atrium (LA) by using a classification neural net to detect results with low accuracy and utilizing an unscented Kalman filter to improve temporal consistency. As far as we know, the effectiveness of temporal consistency has not been explored currently for MYO segmentation of CMR sequences.

### 2.3. Dataset

The automatic cardiac diagnostic challenge (ACDC) dataset consists of both patients with CAD and healthy individuals, whose diagnosis results are extracted from clinical medical cases. There are 150 patients in total and are evenly divided into five subgroups based on their diagnosis results. The five subgroups of patients have systolic heart failure with an infraction, dilated cardiomyopathy, hypertrophic cardiomyopathy, abnormal right ventricle, and no abnormality, respectively. A total of 50 of the patients made up the test dataset on the ACDC website, and the other patients are released as the training dataset. CMR sequences of all patients are collected by two MRI systems with different magnetic strengths (1.5T-Siemens Area, Siemens Medical Solutions, Germany and 3.0T-Siemens Trio Tim, Siemens Medical Solutions, Germany). For each frame in the patient's CMR sequence, there contains a series of short-axis slices covering the LV from base to apex (3). For most patients, the dataset collected 28–40 consecutive frames to cover the whole cardiac cycle. Some of the patients in the dataset may have 5–10% of the cardiac cycle being omitted.

**Figure 2:**
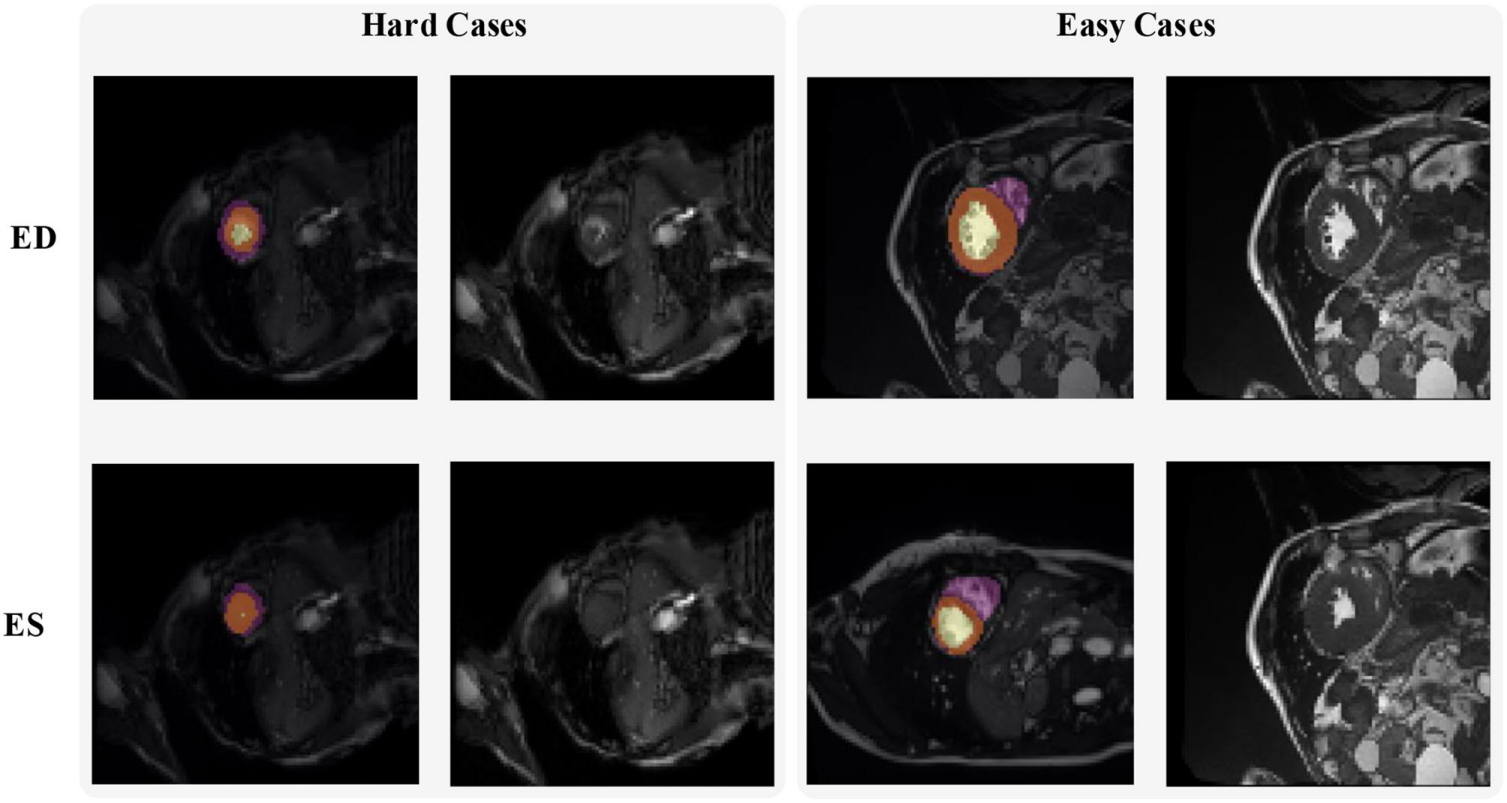
Examples of hard and easy cases of CMR image (slices taken on short-axis). The first and second columns refer to hard cases and the third and fourth columns refer to easy cases.

Figure 2 shows some hard and easy cases in both ED and ES phases of CMR image in the ACDC dataset. The hard cases usually have various characteristics such as low contrast blur image, or extreme anatomical structure. While the easy cases have a high contrast, and less misleading structure with the similar features as LV, RV, and MYO have.

## 3. Methods

**Figure 3:**
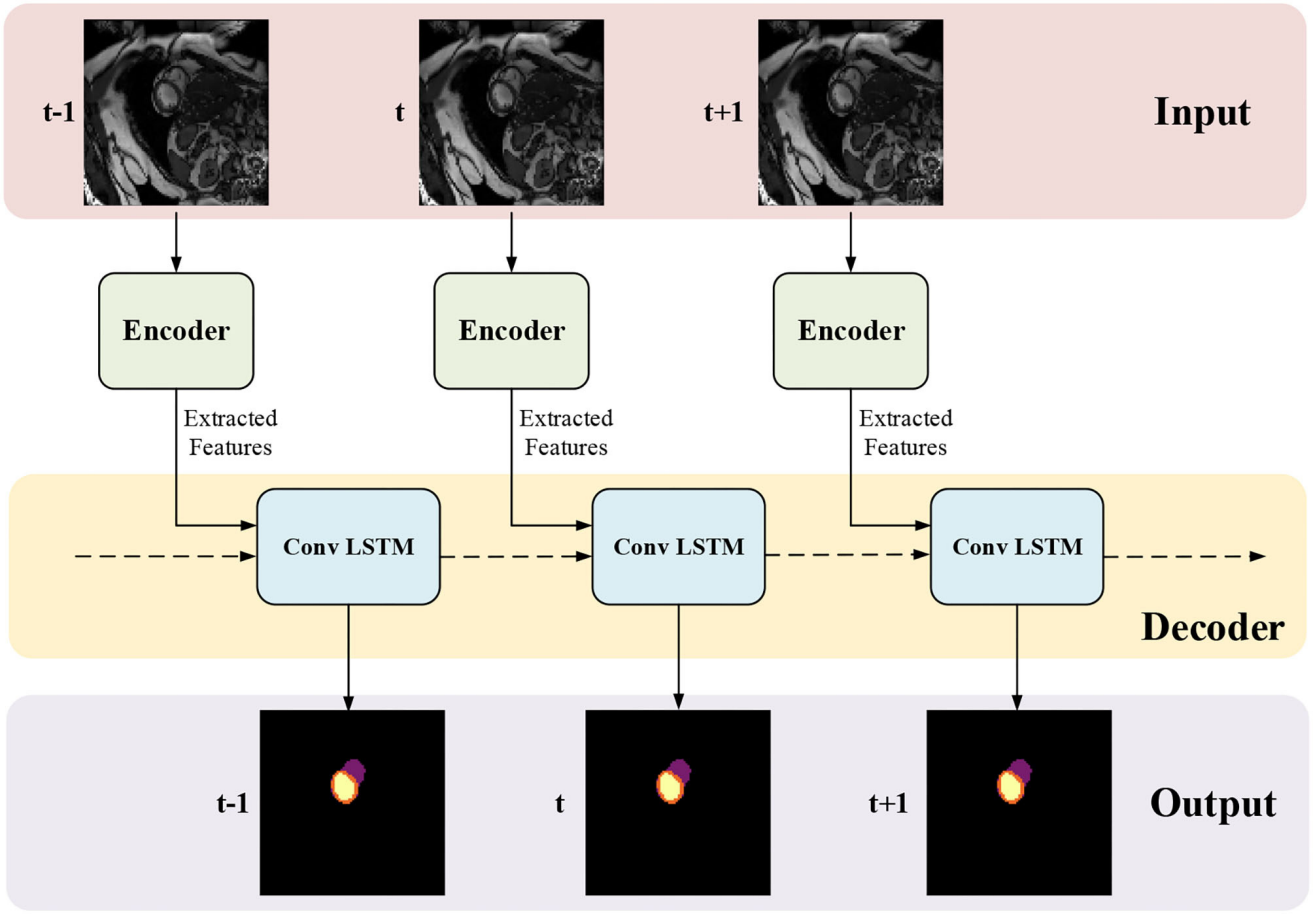
The proposed myocardial (MYO) segmentation architecture, which contains an initial segmentation network (ISN) and a temporal consistency based network (TCN). TSN is based on residual U-net (Res U-Net) (50), while TCN is based on ConvLSTM (51).

The proposed framework for MYO segmentation of CMR image sequences is shown in Figure 3. The input consists of a set of CMR frames from a CMR sequence. The output is the MYO segmentation at the ED and ES phases of the input. Note that the proposed method can accept input sequences with variable lengths by its structural design. Such a feature is designed and ensured in ISN and TCN separately. In ISN, the network will treat each frame of the CMR image sequence as an individual input. In TCN, the nature of RNN allows us to handle the sequence of variable lengths. Therefore, our framework can process input sequences of various lengths.

### 3.1. Initial Segmentation Network

**Figure 4:**
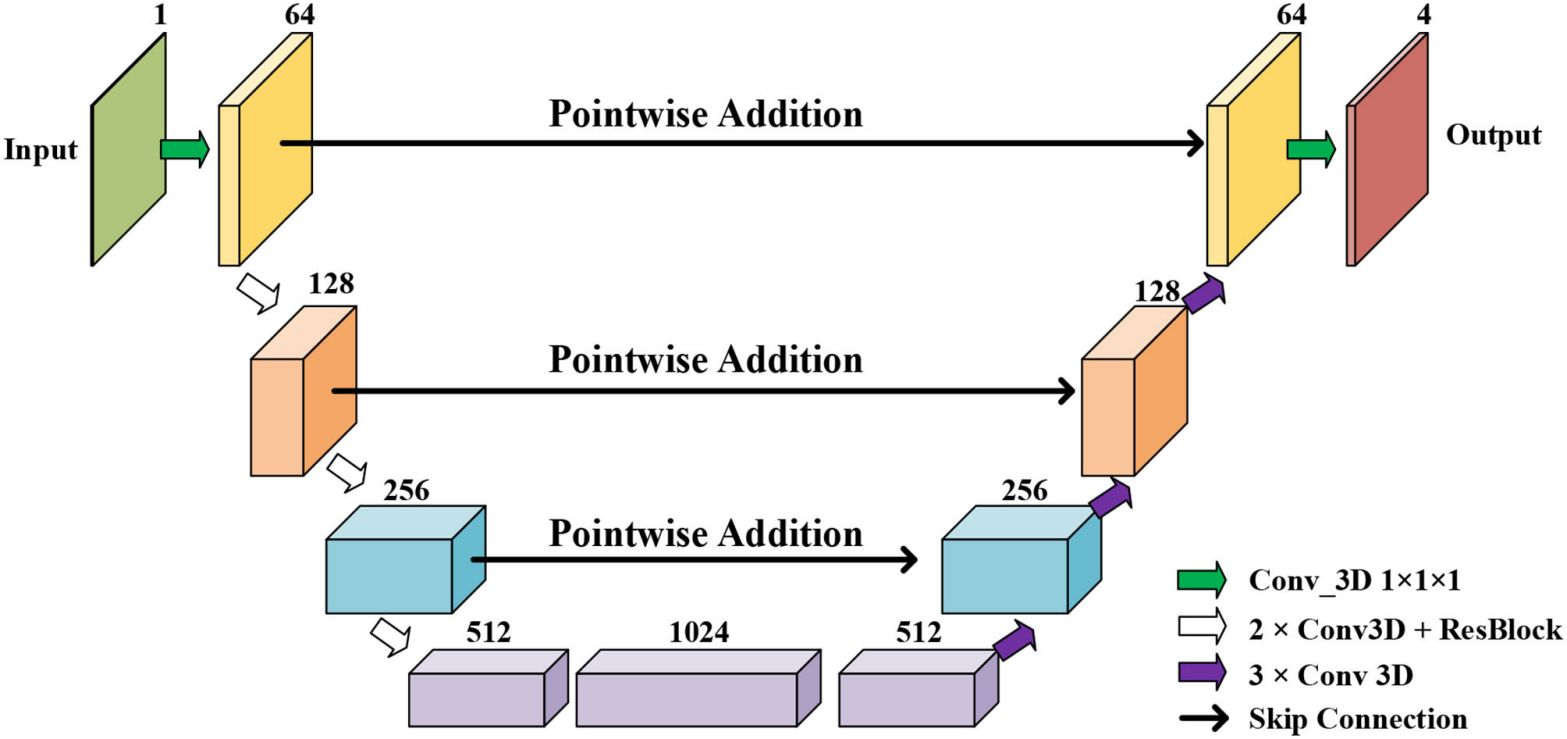
The network structure of our proposed Res U-net based ISN.

Initial Segmentation Network is based on the U-net (50), which is an effective method for a broad range of medical image segmentation tasks. The network structure of ISN is shown in Figure 4. The input of ISN is a single-channel image corresponding to one frame in an MCE sequence. Based on the U-net, we add one residual block between each level of Res-UNet. Also, the results from each layer are combined using pointwise addition instead of concatenation. We expect the addition of residual blocks in the U-net can extract features from the input CMR image without suffering from serious gradient explosion or gradient vanishing problems.

We extract four feature maps from U-net as the output of ISN. Four feature maps obtained from ISN represents the probability that one voxel belongs to the background, LV, RV, and MYO, respectively.

### 3.2. Temporal Consistency Based Network

**Figure 5:**
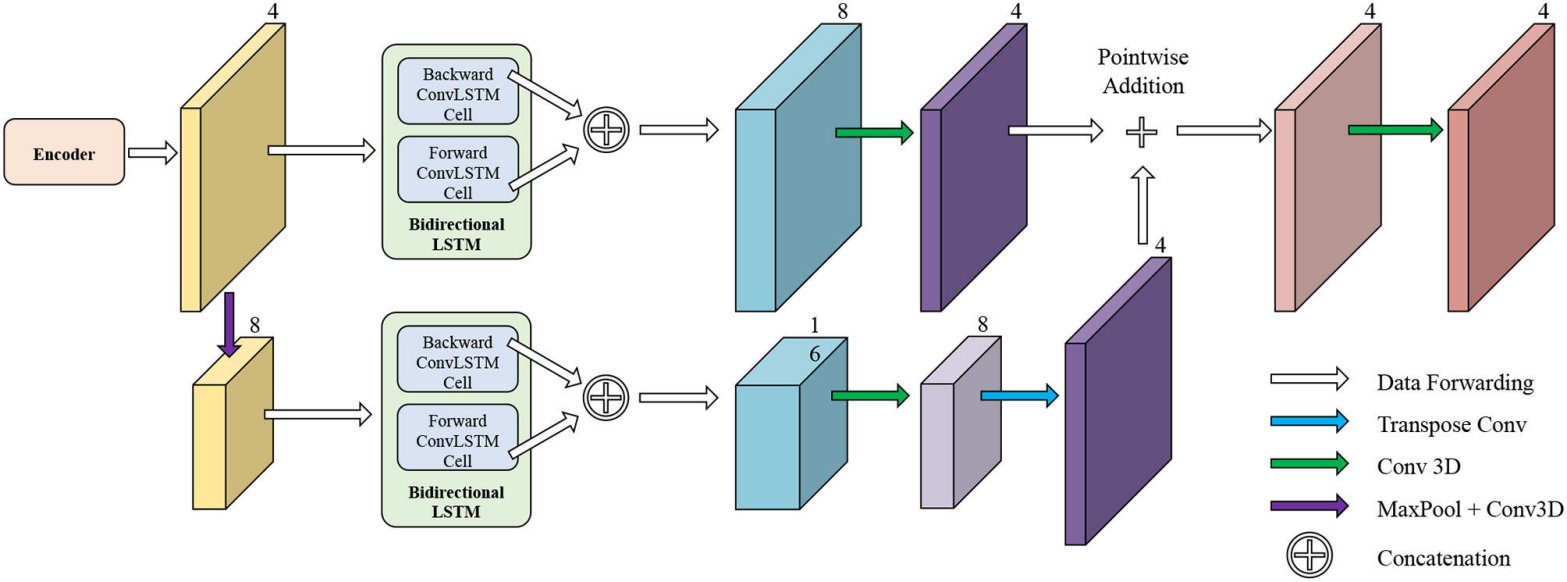
The network structure of TCN. The input of TCN is the features extracted from ISN. TCN consists of hierarchical ConvLSTMs and is able to incorporate temporal information between CMR frames.

The network structure of TCN is shown in Figure 5. It contains a hierarchical recurrent network of ConvLSTMs (52) which acts like a recurrent U-net. The output of TCN is the segmentation result of the myocardium of this frame. The dash arrows in Figure 3 depict the temporal recurrence in TCN. We depict the features extracted by ISN for frame *t* as *f*_*t*_, and *g*_*t,k*_ as the feature for frame *t* in the *k*th level of hierarchical Conv LSTM network and the output of the *k*-th ConvLSTM layer for frame *t* as *y*_*t,k*_. As shown in Equations (1–3), *y*_*t,k*_ depends on three variables: (1) the output of the previous ConvLSTM layer *y*_*t,k*−1_; (2) the extracted features in hierarchical ConvLSTM from ISN *g*_*t,k*_; and (3) the hidden state of the same ConvLSTM layer for the previous frame *y*_*t*−1, *k*_. Note that there is no direct connection between two levels of bi-directional ConvLSTMs in TCN's hierarchical LSTM model. The input of TCN (with 4 channels) will be copied into two copies. The one for level 2 bi-directional ConvLSTM will have 8 channels as the result of an extra 3D Convolution and Max Pooling operations. The one for level 1 bi-directional ConvLSTM will have 4 channels. The outputs from two bi-directional ConvLSTM will be combined by pointwise addition.


(1) $$\begin{array}{l} h_{input}=\left[g_{t,k}\;|\;y_{t,k-1}\right] \end{array}$$

(2) $$\begin{array}{l} h_{state}=y_{t-1,k} \end{array}$$

(3) $$\begin{array}{l} y_{t,k}=ConvLSTM_{k}\left(h_{input},h_{state}\right) \end{array}$$

In Equation (3), *h*_*input*_ is the input of ConvLSTM, and *h*_*state*_ is the hidden state input of ConvLSTM. [*A*|*B*] is the concatenate operation for tensors *A* and *B* on the feature axis. For the first frame of a CMR sequence, *h*_*state*_ is a matrix of ones, which means no prior information is known.

### 3.3. Bi-Directional Training

**Figure 6:**
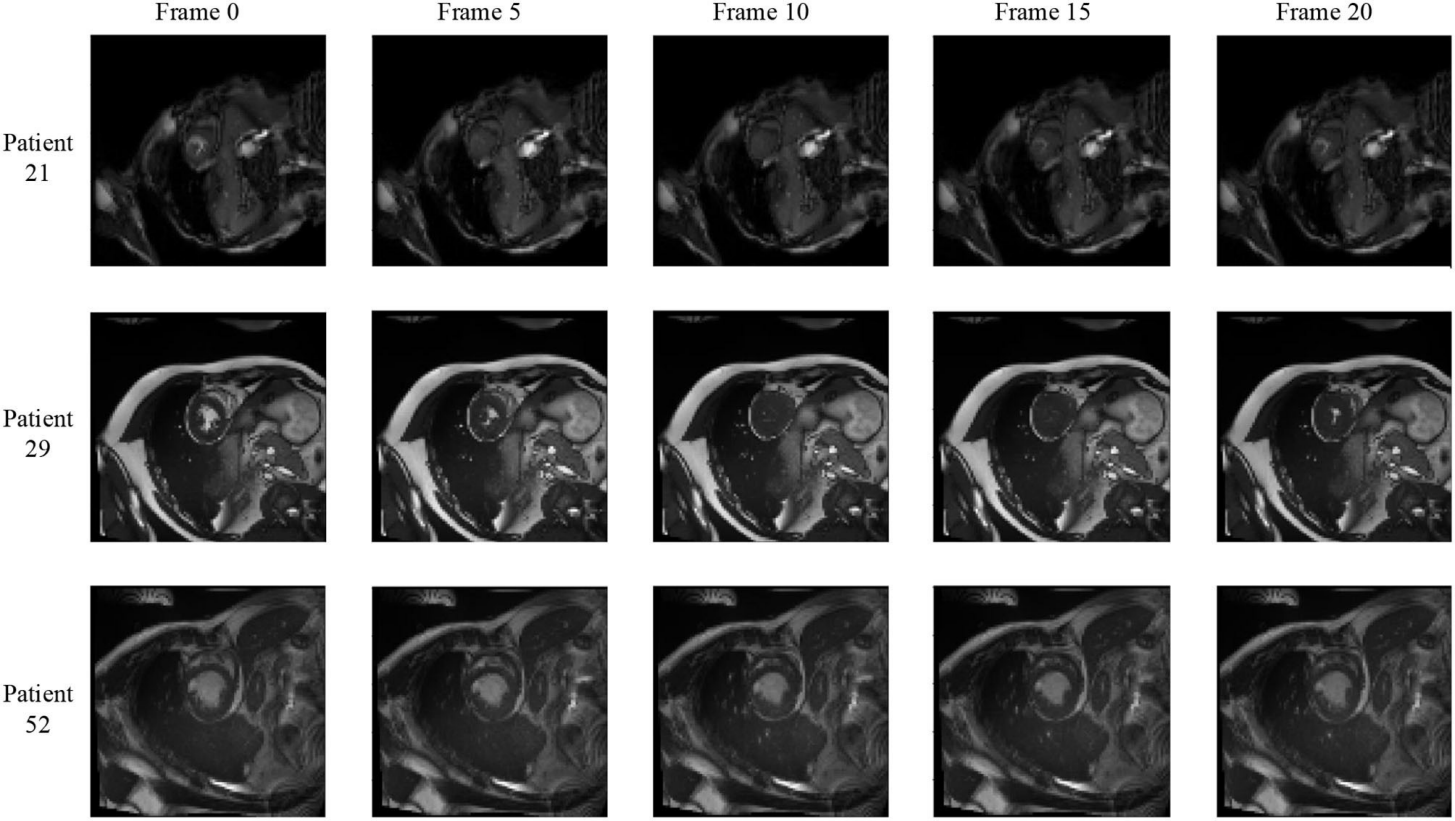
Frames of CMR sequence from three patients. Note the brightness heterogeneity in left ventricular (LV) and right ventricular (RV) on the first few frames. Using LSTM in TCN, the model can get more temporal information from the previous and future frames and result in more accurate segmentation.

We notice that the prediction results of myocardium segmentation in the CMR image are highly related to the segmentation result of frames either behind or after it. The first frame of the CMR sequence will not receive enough information if we only use forward Conv LSTM. Figure 6 shows some frames of the CMR images of different patients in the ACDC dataset. We can see that the frames of CMR image of the last frame are highly related to the image of the next frame. Consequently, the prediction error of the first frame due to the brightness heterogeneity may propagate to the rest of the CMR frames.

**Figure 7:**
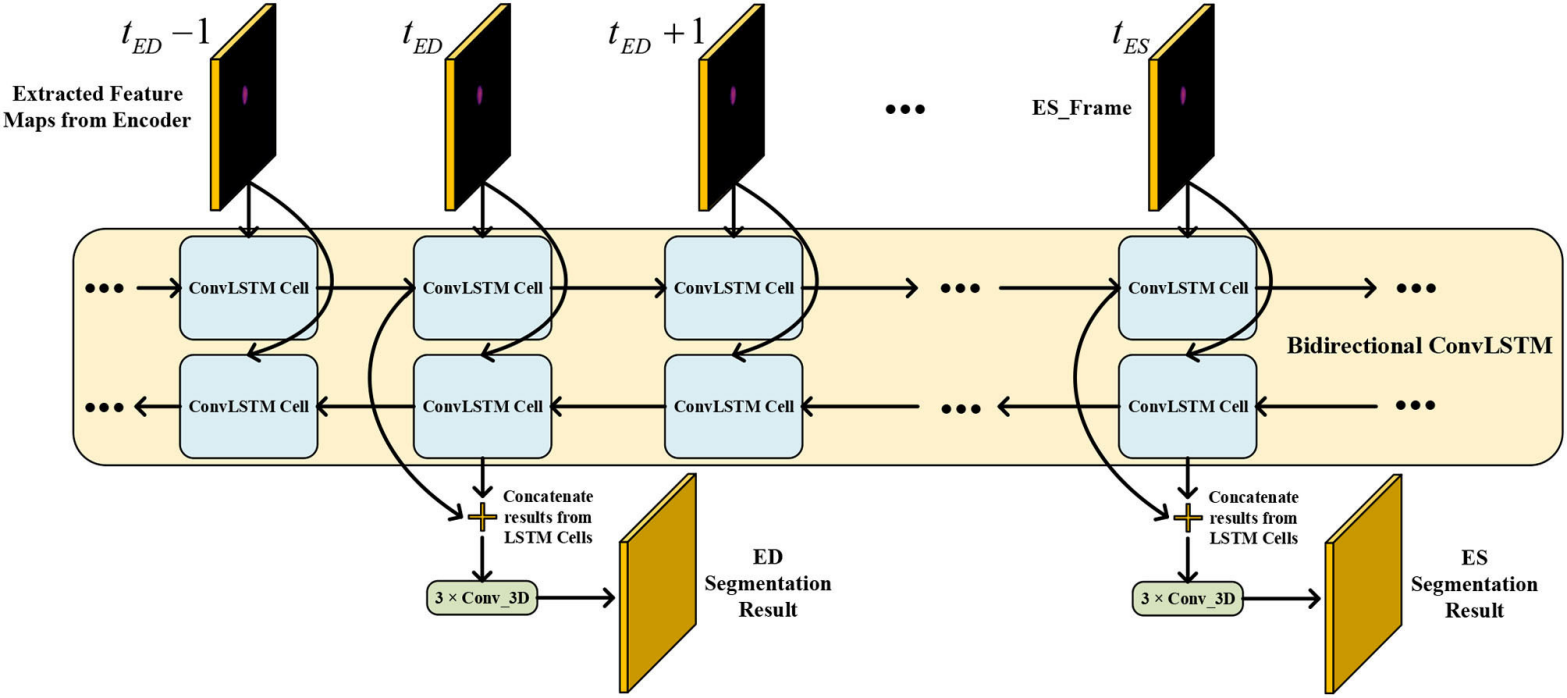
Illustration of our proposed bi-direction training approach.

Therefore, we adopted a bi-directional training approach to alleviate this problem. Specifically, we used two Conv LSTMs in our TCN model. One will propagate forward, from frame 1 to frame *T*, while the other will propagate backward, from frame *T* to frame 1. *T* is the total number of frames in one CMR sequence. Figure 7 presents the workflow of the proposed bi-directional training approach. The Forward ConvLSTM cell at time *t* takes in two inputs: the cell state at time *t*−1 and the ISN's output for a frame of CMR image at time *t*. The Backward ConvLSTM cell at time *t* also takes in two inputs: the cell state at time *t*+1 and ISN's output for a frame of CMR image at time *t*. When the ConvLSTM cell is initialized, the cell's hidden state is initialized as a zero vector. Such an approach can better exploit the temporal information along with the frames, thus, improving the segmentation accuracy.

Note that our method is trained in two phases. In the first phase, ISN is trained. ISN's parameters are updated using the ground truth label from ACDC dataset frames at ED and ES. In the second phase, ISN and TCN are trained together. The supervision signal will only enter the framework from the output of TCN. The computation graph of ISN and TCN are connected, and the gradient can flow from TCN to ISN properly. TCN will give out segmentation result for each frame of the CMR sequence. Among all results, we will only take out the frame at ED and ES time for lost function calculation.

## 4. Experiments

### 4.1. Experiment Setup

In this section, we evaluated the performance of our proposed framework in the MYO segmentation task of CMR sequences. The residual U-net (Res U-net) implementation is used as our baseline. We compared the proposed framework (Res U-net+ConvLSTM) and Res U-net. Res U-net and ConvLSTM are implemented using PyTorch based on Xingjian et al. (52) and Isensee et al. (53) separately. The CMR images are resampled into 96 × 96 × 24 using the linear resample method. For data augmentation during training, we scaled all images by 0.8 and 1.2 and flipped them on the *x*-axis and *y*-axis, respectively. During testing and validation, we did not employ any augmentations. For each iteration, a complete CMR sequence containing 28–40 frames of a patient was used for training. Due to the limited GPU memory, the batch size is set to 1, which means in each iteration, 1 CMR sequence containing 28–40 frames is fed for training. We trained ISN for 10 epochs with a learning rate of 0.0001. Then, we trained ISN and TCN together with a learning rate of 0.0001 and a learning rate decay of 0.7 per epoch for another 10 epochs. Three loss functions including soft Dice loss, cross-entropy loss, and contour loss (54) are used for training. The adaptive momentum estimation (ADAM) optimizer is adopted to update the parameters.

We split the ACDC dataset into the training set, validation set, and testing set by a ratio of 7:2:1 based on the patient number. Therefore, there are 70 patients in the training set, 20 patients in the validation set, and 10 patients in the testing set. Dice coefficient and Inter-section over Union (IoU) are used to evaluate the segmentation performance, which is defined as:


(4) $$\begin{array}{ll} Dice\left(P,T\right) & =2\times \frac{\sum_{n=1}^{N}\left(P_{n}\times T_{n}\right)}{\sum_{n=1}^{N}\left(P_{n}+T_{n}\right)} \end{array}$$

(5) $$\begin{array}{ll} IoU\left(P,T\right) & =\frac{\sum_{n=1}^{N}\left(P_{n}\times T_{n}\right)}{\sum_{n=1}^{N}\left(P_{n}+T_{n}-P_{n}\times T_{n}\right)} \end{array}$$

in which *P* and *T* refer to the prediction and ground truth mask, respectively. *n* is the index of all voxels (totally *N* voxels).

### 4.2. Results and Discussion

Table 1 shows the results of MYO segmentation for LV, RV, and MYO at the ED phase and ES phase. Dice coefficient on each label class and IoU are reported. Res U-net+f-ConvLSTM refers to training the proposed framework forwardly from frame 0 to frame *T* while our framework (Res U-net+bi-ConvLSTM) refers to training in bi-direction. We noticed that our framework outperforms baseline implementation in all metrics for both ED and ES frames. Specifically, our Res U-net+f-ConvLSTM implementation has an improvement of 0.01, 0.10, −0.81, 0.61, 0.55, and 0.30% on the Dice coefficient of LV, RV, and MYO at ED frame and ES frame, respectively. The experiment results of Res U-net+f-ConvLSTM and our framework show that by adding a backward training step, we can further increase the segmentation performance. Our framework's implementation has an improvement of 1.11, 0.64, 0.82, 0.83, 2.39, and 1.02% on the Dice coefficient of LV, RV, and MYO at ED frame and ES frame, respectively.

**Table 1:** Comparison of our proposed framework against residual U-net (Res U-net).

	**ED**					**ES**				
	**Dice**			**Overall**		**Dice**			**Overall**	
	**LV**	**RV**	**MYO**	**IoU**	**p-value**	**LV**	**RV**	**MYO**	**IoU**	**p-value**
Res U-net	0.8856	0.8073	0.7178	0.5583 ± 0.0045	<0.0001	0.8050	0.6841	0.7554	0.4053 ± 0.0379	<0.0001
Res U-net + f-ConvLSTM	0.8857	0.8082	0.7097	0.5586 ± 0.0044	<0.0001	0.8056	0.6896	0.7588	0.4186 ± 0.0038	<0.0001
Our framework	**0.8967**	**0.8146**	**0.7260**	**0.5587 ± 0.0045**	–	**0.8133**	**0.7080**	**0.7656**	**0.4231 ± 0.0038**	–

*Res U-net+f-ConvLSTM refers to forward ConvLSTM that is trained forwardly from frame 1 to frame T. Res U-net+bi-ConvLSTM (our framework) refers to training both forward ConvLSTM and backward ConvLSTM, where backward ConvLSTM processes input CMR images from frame T to frame 1. Two-sided t-test with 95% CI is used for statistic analysis. The bold values correspond to the optimal performance*.

**Figure 8:**
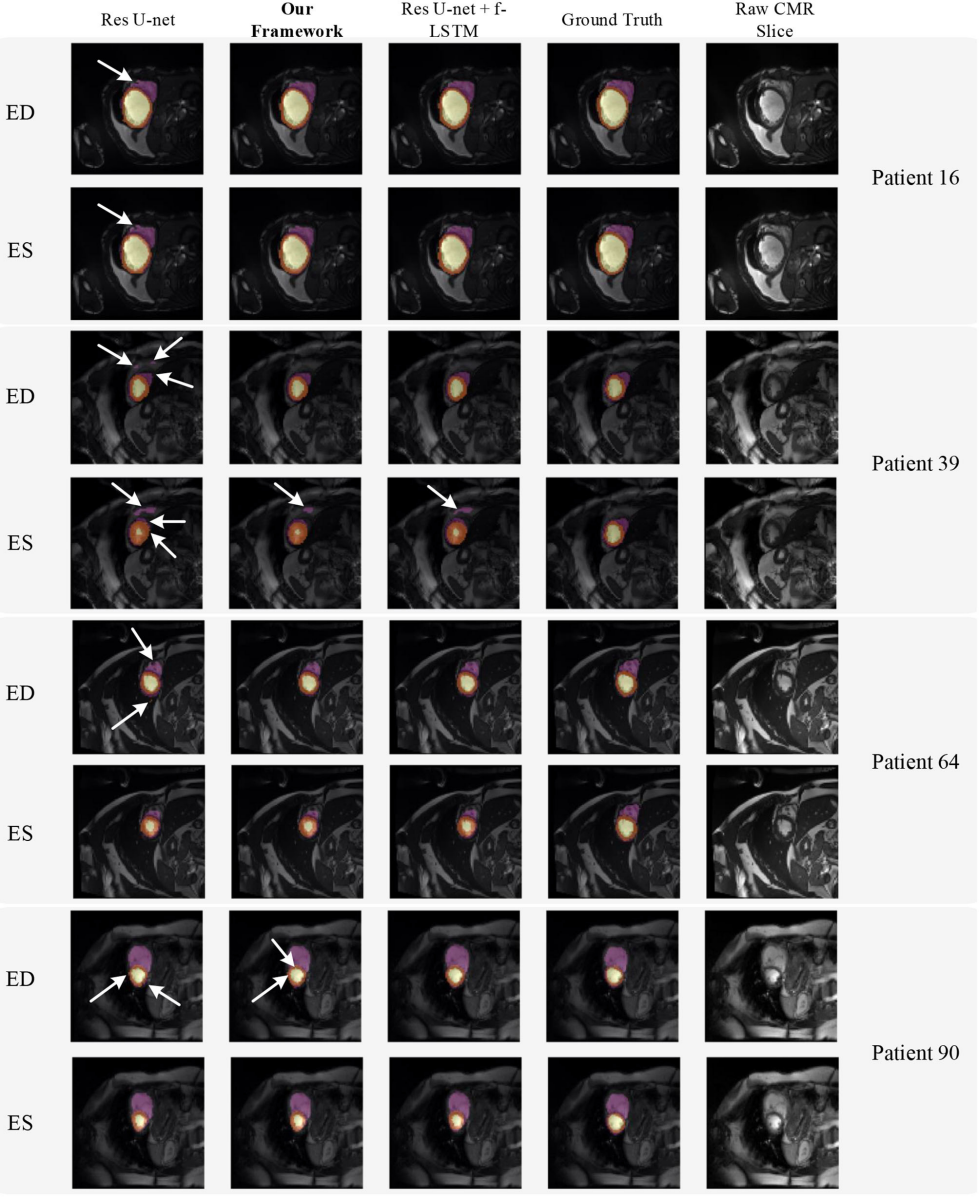
Visualization of CMR image segmentation results of three different patients in both end-diastolic (ED) phase and end-systolic (ES) phase. Yellow, orange, and purple areas refer to the LV, myocardial (MYO), and RV, respectively. Each row refers to the segmentation result of Res U-net, our framework, Res U-net + f-ConvLSTM, and ground truth from left to right, respectively. The white arrows in the image specifically point out the segmentation result that is inconsistent. We can see the f-ConvLSTM and bi-ConvLSTM model, which incorporates the temporal information between frames can greatly decrease the existence of such inconsistent segmentation results. Also, most errors in Res U-net + fConvLSTM and our framework are in hard cases like Patient 39, where the input CMR image has low contrast and vague contour between labeled tissue and background tissue.

Figure 8 shows the visualization of segmentation results of four different patients (Patient 16, Patient 39, Patient 64, and Patient 90) in both ED phase and ES phase by Res U-net, our framework, and Res U-net+f-ConvLSTM. Each row refers to the result of Res U-net, our framework, Res U-net+f-ConvLSTM, ground truth, and raw CMR slice, respectively. From Figure 8, we can see the f-ConvLSTM and bi-ConvLSTM, which has temporal consistency between frames, have less inconsistent segmentation result as marked by the white arrow in the figure. However, for few cases, the temporal consistency may not eliminate the inconsistency in the segmentation result completely. This happens when a stable feature on the CMR image is recognized as an incorrect label in the framework. Since the misleading structure will remain on all the CMR frames in the sequence, the temporal consistency provided by LSTM will not be able to remove such an inconsistency. Table 2 demonstrates the quantitative results for the ED phase and ES phase of Patients 16, 39, 64, and 90 corresponding to Figure 8. We can see that our proposed framework tends to predict more consistently and accurately than the baseline, especially in the first few frames such as the ES phase of Patient 16, where the obvious defect exists in the segmentation result. Comparing Res U-net+f-ConvLSTM and our framework on segmentation boundaries, we can observe that the bi-directional training can help our framework to produce more consistent results in most cases. Although in some cases the segmentation of Res U-net+f-ConvLSTM is better than that of our framework, this might be caused by the constraint from backward temporal information that makes the segmentation lack flexibility. The overall performance of our framework is superior in terms of all the metrics. It can be seen that the Dice coefficients of the ED phase are usually higher than the ES phase. However, our framework can achieve higher performance on both phases compared with Res U-net implementation.

**Table 2:** Quantitative segmentation results of different models for frames in Figure 8.

**Patient ID**		**Res U-net**		**Res U-net+f-ConvLSTM**		**Our framework**	
		**IoU**	**Dice**	**IoU**	**Dice**	**IoU**	**Dice**
Patient 16	ED	0.5417	0.8245	0.5417	0.8433	0.5417	0.8499
	ES	0.5729	0.8168	0.5729	0.8196	0.5729	0.8234
Patient 39	ED	0.6771	0.8132	0.6771	0.8364	0.6771	0.8348
	ES	0.7396	0.7556	0.7396	0.7688	0.7396	0.7587
Patient 64	ED	0.6666	0.8537	0.6667	0.8653	0.6667	0.8616
	ES	0.6875	0.8334	0.6875	0.8368	0.6875	0.8347
Patient 90	ED	0.6563	0.8099	0.6563	0.8027	0.6563	0.8043
	ES	0.6563	0.7476	0.6563	0.7482	0.6563	0.7623

Note that the work (18) used the class-balanced loss and transfer learning to improve the performance of Res 3D U-net on the ACDC dataset. They achieved Dice coefficients of 0.864, 0.789, 0.775, and 0.770 on segmentation of LV and RV in the ED phase and ES phase, respectively, while our framework achieves higher Dice coefficients of 0.897, 0.815, 0.813, and 0.708, respectively.

## 5. Discussion

**Figure 9:**
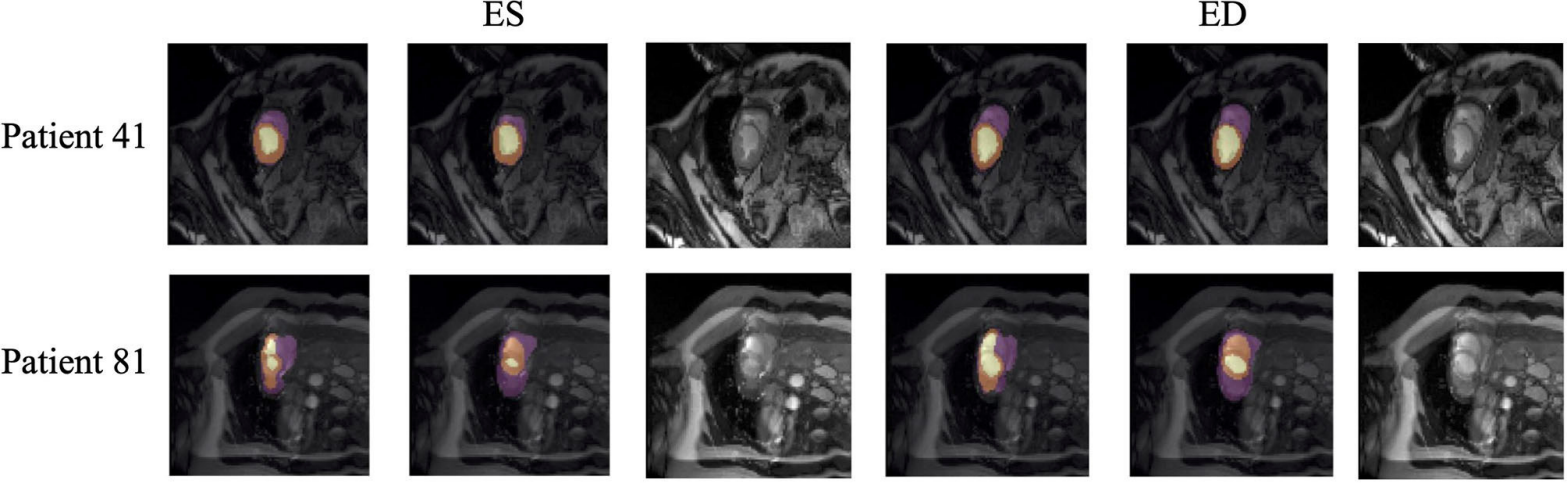
Typical segmentation error of our framework. The image on Columns 1 and 4 are the segmentation results and images on Columns 2 and 5 are the corresponding ground truth. The segmentation error is usually caused by brightness heterogeneity, lack of contrast, or the improper input image due to faulty setup of magnetic resonance system or the misoperations of operators. The white arrows in the image specifically point out the segmentation result that is inconsistent.

Quantitative segmentation results and grading results demonstrate the superiority of our framework compared with the Res U-net baseline implementation. However, there are still some cases where our framework cannot predict reasonable boundaries. For example, Figure 9A shows the segmentation results of Patient 41 in ES and ED phases by our framework. We can notice that there exists deviation between the ground truth boundary (images on Columns 2 and 4) and the prediction boundary (images on Columns 1 and 3). This is because the flow of blood in the RV cavity leads to the brightness heterogeneity in the RV area of the CMR image, which makes the image intensity of the ground truth RV region similar to the surrounding cardiac structures (e.g., heart chambers), and finally leads to segmentation failure.

There are some cases as shown in Figure 9B in which CMR sequences have serious defects and ghosting. This may be caused by the improper setup of the magnetic resonance system or the mistake of operators. Therefore, it is hard for our framework to find a plausible MYO boundary even though our framework is able to correct some segmentation errors based on temporal information between frames.

Note that compared with existing works for MYO segmentation of CMR sequences, our main contribution is to adopt temporal consistency for coronary artery disease (CAD) diagnosis which has not been extensively explored before. The results show that temporal consistency is important in the MYO segmentation of CMR sequences. Compared with existing works making use of temporal consistency, our methodology differs from the others as follows. Yan et al. (13) incorporated an optical flow field to capture the temporal consistency, while (14) adopted a moving mesh correspondence algorithm to refine segmentation using temporal consistency. Qin et al. (15) and Guo et al. (16) both proposed a motion estimation branch to assist the segmentation. Ours proposed a two-stage segmentation framework in which conventional neural networks and recurrent neural networks are combined to incorporate temporal information between sequences to ensure temporal consistency. In addition, ours additionally adopt contour loss (54) to facilitate the learning process.

## 6. Conclusion

In this article, we proposed an MYO segmentation framework of CMR sequences for CAD diagnosis. Specifically, we proposed to combine conventional neural networks and recurrent neural networks to incorporate temporal information between sequences to ensure temporal consistency. Extensive experiments showed that compared with Res U-net, our proposed framework can achieve an improvement of 1 to 2% in Dice coefficient. In addition, we introduced a bi-directional training approach to further reduce segmentation error introduced by the first few frames in the forward training process. Experiment results demonstrate that our bi-directional training approach can further improve the segmentation performance.

## Data Availability Statement

The original contributions presented in the study are included in the article/supplementary material, further inquiries can be directed to the corresponding author/s.

## Author Contributions

YC, WX, JZha, and HQ contributed to data collection, analysis, and experiment. DZ, YS, HY, JZhu, YD, and QJ contributed to discussion and writing. YZ, MH, and XX contributed to project planning, development, discussion, and writing. All authors contributed to the article and approved the submitted version.

## Funding

This study was supported by the National Key Research and Development Program of China (No. 2018YFC1002600), the Science and Technology Planning Project of Guangdong Province, China (Nos. 2017B090904034, 2017B030314109, 2018B090944002, and 2019B020230003), Guangdong Peak Project (No. DFJH201802), and the National Natural Science Foundation of China (No. 62006050).

## Conflict of Interest

The authors declare that the research was conducted in the absence of any commercial or financial relationships that could be construed as a potential conflict of interest.

## Publisher's Note

All claims expressed in this article are solely those of the authors and do not necessarily represent those of their affiliated organizations, or those of the publisher, the editors and the reviewers. Any product that may be evaluated in this article, or claim that may be made by its manufacturer, is not guaranteed or endorsed by the publisher.

## References

[1] Vos T Allen C Arora M Barber RM Bhutta ZA Brown A. Global, regional, and national incidence, prevalence, and years lived with disability for 310 diseases and injuries, 1990–2015: a systematic analysis for the global burden of disease study 2015. Lancet. (2016) 388:1545–602. 10.1016/S0140-6736(16)31678-6 27733282 PMC5055577

[2] Malakar AK Choudhury D Halder B Paul P Uddin A Chakraborty S. A review on coronary artery disease, its risk factors, and therapeutics. J Cell Physiol. (2019) 234:16812–23. 10.1002/jcp.28350 30790284

[3] Bernard O Lalande A Zotti C Cervenansky F Yang X Heng P. Deep learning techniques for automatic MRI cardiac multi-structures segmentation and diagnosis: is the problem solved? IEEE Trans Med Imag. (2018) 37:2514–25. 10.1109/TMI.2018.2837502 29994302

[4] Wang T Xiong J Xu X Jiang M Yuan H Huang M. MSU-Net: Multiscale statistical U-net for real-time 3D cardiac MRI video segmentation. In: International Conference on Medical Image Computing and Computer-Assisted Intervention. Springer; 2019. p. 614–622.

[5] Wang T Xu X Xiong J Jia Q Yuan H Huang M. Ica-unet: Ica inspired statistical unet for real-time 3d cardiac cine mri segmentation. In: International Conference on Medical Image Computing and Computer-Assisted Intervention. Lima: Springer (2020). p. 447–57.

[6] Odden MC Coxson PG Moran A Lightwood JM Goldman L Bibbins-Domingo K. The impact of the aging population on coronary heart disease in the United States. Am J Med. (2011) 124:827–33. 10.1016/j.amjmed.2011.04.010 21722862 PMC3159777

[7] Li Y Ho CP Chahal N Senior R Tang MX. Myocardial segmentation of contrast echocardiograms using random forests guided by shape model. In: International Conference on Medical Image Computing and Computer-Assisted Intervention. Athens: Springer (2016). p. 158–65.

[8] Guo Y Du GQ Xue JY Xia R Wang Yh. A novel myocardium segmentation approach based on neutrosophic active contour model. Comput Methods Prog Biomed. (2017) 142:109–16. 10.1016/j.cmpb.2017.02.020 28325439

[9] Barbosa D Dietenbeck T Heyde B Houle H Friboulet D D'hooge J. Fast and fully automatic 3-d echocardiographic segmentation using B-spline explicit active surfaces: feasibility study and validation in a clinical setting. Ultrasound Med Biol. (2013) 39:89–101. 10.1016/j.ultrasmedbio.2012.08.008 23200179

[10] van Stralen M Leung K Voormolen MM de Jong N van der Steen AF Reiber JH. Time continuous detection of the left ventricular long axis and the mitral valve plane in 3-D echocardiography. Ultrasound Med Biol. (2008) 34:196–207. 10.1016/j.ultrasmedbio.2007.07.016 17935871

[11] Leclerc S Grenier T Espinosa F Bernard O. A fully automatic and multi-structural segmentation of the left ventricle and the myocardium on highly heterogeneous 2D echocardiographic data. In: 2017 IEEE International Ultrasonics Symposium (IUS). Washington, DC: IEEE (2017). p. 1–4.

[12] Chen H Zheng Y Park JH Heng PA Zhou SK. Iterative multi-domain regularized deep learning for anatomical structure detection and segmentation from ultrasound images. In: International Conference on Medical Image Computing and Computer-Assisted Intervention. Athens: Springer (2016). p. 487–95.

[13] Yan W Wang Y van der Geest RJ Tao Q. Cine MRI analysis by deep learning of optical flow: adding the temporal dimension. Comput. Biol. Med. (2019) 111:103356. 10.1016/j.compbiomed.2019.103356 31323604

[14] Punithakumar K Tahmasebi N Boulanger P Noga M. Convolutional neural network based automated RV segmentation for hypoplastic left heart syndrome MRI. In: 8th International Conference of Pattern Recognition Systems (ICPRS 2017). IET (2017). p. 1–6.

[15] Qin C Bai W Schlemper J Petersen SE Piechnik SK Neubauer S. Joint learning of motion estimation and segmentation for cardiac MR image sequences. In: International Conference on Medical Image Computing and Computer-Assisted Intervention. Springer (2018). p. 472–80.

[16] Guo Y Bi L Zhu Z Feng DD Zhang R Wang Q. Automatic left ventricular cavity segmentation via deep spatial sequential network in 4D computed tomography. Comput Med Imag Graph. (2021) 91:101952. 10.1016/j.compmedimag.2021.101952 34144318

[17] Hernandez KAL Rienmüller T Baumgartner D Baumgartner C. Deep learning in spatiotemporal cardiac imaging: a review of methodologies and clinical usability. Comput Biol Med. (2021) 130:104200. 10.1016/j.compbiomed.2020.104200 33421825

[18] Yang X Bian C Yu L Ni D Heng PA. Class-balanced deep neural network for automatic ventricular structure segmentation. In: Lecture Notes in Computer Science. Cham: Springer International Publishing (2018). p. 152–160.

[19] Xu X Lu Q Yang L Hu S Chen D Hu Y. Quantization of fully convolutional networks for accurate biomedical image segmentation. In: Proceedings of the IEEE Conference on Computer Vision and Pattern Recognition. Salt Lake City, UT (2018). p. 8300–8.

[20] Xu X Ding Y Hu SX Niemier M Cong J Hu Y. Scaling for edge inference of deep neural networks. Nat Electron. (2018) 1:216–22. 10.1038/s41928-018-0059-3

[21] Xu X Wang T Shi Y Yuan H Jia Q Huang M. Whole heart and great vessel segmentation in congenital heart disease using deep neural networks and graph matching. In: International Conference on Medical Image Computing and Computer-Assisted Intervention. Shenzhen: Springer (2019). p. 477–85.

[22] Xu X Wang T Zhuang J Yuan H Huang M Cen J. Imagechd: a 3d computed tomography image dataset for classification of congenital heart disease. In: International Conference on Medical Image Computing and Computer-Assisted Intervention. Lima: Springer (2020). p. 77–87.

[23] Xu X Qiu H Jia Q Dong Y Yao Z Xie W. AI-CHD: an AI-based framework for cost-effective surgical telementoring of congenital heart disease. Commun ACM. (2021) 64:66–74. 10.1145/3450409

[24] Zhang J Zhang Y Zhu S Xu X. Constrained multi-scale dense connections for accurate biomedical image segmentation. In: 2020 IEEE International Conference on Bioinformatics and Biomedicine (BIBM). Seoul: IEEE (2020). p. 877–84.

[25] Liu Z Xu X Liu T Liu Q Wang Y Shi Y. Machine vision guided 3d medical image compression for efficient transmission and accurate segmentation in the clouds. In: Proceedings of the IEEE/CVF Conference on Computer Vision and Pattern Recognition. Long Beach, CA (2019). p. 12687–96.

[26] Zhang J Zhang Y Xu X. Pyramid U-Net for retinal vessel segmentation. In: ICASSP 2021-2021 IEEE International Conference on Acoustics, Speech and Signal Processing (ICASSP). Toronto, ON: IEEE (2021). p. 1125–9.

[27] Tobon-Gomez C Butakoff C Aguade S Sukno F Moragas G Frangi AF. Automatic construction of 3D-ASM intensity models by simulating image acquisition: Application to myocardial gated SPECT studies. IEEE Trans Med Imag. (2008) 27:1655–67. 10.1109/TMI.2008.2004819 18955180

[28] Cootes TF Taylor CJ Cooper DH Graham J. Active shape models-their training and application. Comput Vis Image Understand. (1995) 61:38–59. 10.1006/cviu.1995.1004

[29] Cootes TF Edwards GJ Taylor CJ. Active appearance models. IEEE Trans Pattern Anal Mach Intell. (2001) 23:681–685. 10.1109/34.927467

[30] Ordas S Boisrobert L Huguet M Frangi A. Active shape models with invariant optimal features (IOF-ASM) application to cardiac MRI segmentation. In: Comput Cardiol Volume 30. Thessaloniki (2003) p. 633–6.

[31] van Assen HC Danilouchkine MG Frangi AF Ordás S Westenberg JJM Reiber JHC. SPASM: a 3D-ASM for segmentation of sparse and arbitrarily oriented cardiac MRI data. Med Image Anal. (2006) 10:286–303. 10.1016/j.media.2005.12.001 16439182

[32] Tobon-Gomez C Sukno FM Butakoff C Huguet M Frangi AF. Automatic training and reliability estimation for 3D ASM applied to cardiac MRI segmentation. Phys Med Biol. (2012) 57:4155–74. 10.1088/0031-9155/57/13/4155 22683992

[33] Santiago C Nascimento JC Marques JS. A new ASM framework for left ventricle segmentation exploring slice variability in cardiac MRI volumes. Neural Comput Appl. (2016) 28:2489–500. 10.1007/s00521-016-2337-1

[34] Zhang L Gooya A Dong B Hua R Petersen SE Medrano-Gracia P. Automated quality assessment of cardiac MR images using convolutional neural networks. In: Simulation and Synthesis in Medical Imaging. Athens: Springer International Publishing (2016). p. 138–45.

[35] Poudel RPK Lamata P Montana G. Recurrent fully convolutional neural networks for multi-slice MRI cardiac segmentation. In: Reconstruction, Segmentation, and Analysis of Medical Images. Springer (2016). p. 83–94.

[36] Simantiris G Tziritas G. Cardiac MRI segmentation with a dilated CNN incorporating domain-specific constraints. IEEE J Sel Top Signal Process. (2020) 14:1235–43. 10.1109/JSTSP.2020.3013351

[37] Isensee F Jaeger PF Full PM Wolf I Engelhardt S Maier-Hein KH. Automatic cardiac disease assessment on cine-MRI via time-series segmentation and domain specific features. In: Lecture Notes in Computer Science. Cham: Springer International Publishing (2018). p. 120–9.

[38] Simantiris G Tziritas G. Cardiac MRI segmentation with a dilated CNN incorporating domain-specific constraints. IEEE J Sel Top Signal Process. (2020) 14:1235–43.

[39] Zotti C Luo Z Lalande A Jodoin PM. Convolutional neural network with shape prior applied to cardiac MRI segmentation. IEEE J Biomed Health Inf. (2019) 23:1119–28. 10.1109/JBHI.2018.2865450 30113903

[40] Painchaud N Skandarani Y Judge T Bernard O Lalande A Jodoin PM. Cardiac MRI segmentation with strong anatomical guarantees. In: Lecture Notes in Computer Science. Shenzhen: Springer International Publishing (2019). p. 632–40.

[41] Khened M Alex V Krishnamurthi G. Densely connected fully convolutional network for short-axis cardiac cine MR image segmentation and heart diagnosis using random forest. In: Lecture Notes in Computer Science. Cham: Springer International Publishing (2018). p. 140–51.

[42] Baumgartner CF Koch LM Pollefeys M Konukoglu E. An exploration of 2D and 3D deep learning techniques for cardiac MR image segmentation. In: Lecture Notes in Computer Science. Cham: Springer International Publishing (2018). p. 111–19.

[43] Calisto MB Lai-Yuen SK. AdaEn-Net: an ensemble of adaptive 2D–3D Fully Convolutional Networks for medical image segmentation. Neural Netw. (2020) 126:76–94. 10.1016/j.neunet.2020.03.007 32203876

[44] Wolterink JM Leiner T Viergever MA Išgum I. Automatic segmentation and disease classification using cardiac cine MR images. In: Lecture Notes in Computer Science. Springer International Publishing (2018). p. 101–10.

[45] Rohé MM Sermesant M Pennec X. Automatic multi-atlas segmentation of myocardium with SVF-net. In: Lecture Notes in Computer Science. Québec: Springer International Publishing (2018). p. 170–77.

[46] Zotti C Luo Z Humbert O Lalande A Jodoin PM. GridNet with automatic shape prior registration for automatic MRI cardiac segmentation. In: Lecture Notes in Computer Science. Québec: Springer International Publishing (2018). p. 73–81.

[47] Patravali J Jain S Chilamkurthy S. 2D-3D fully convolutional neural networks for cardiac MR segmentation. In: Lecture Notes in Computer Science. Québec: Springer International Publishing (2018). p. 130–9.

[48] Wei H Cao H Cao Y Zhou Y Xue W Ni D. Temporal-consistent segmentation of echocardiography with co-learning from appearance and shape. In: International Conference on Medical Image Computing and Computer-Assisted Intervention. Lima: Springer (2020). p. 623–32.

[49] Zhang X Noga M Martin DG Punithakumar K. Fully automated left atrium segmentation from anatomical cine long-axis MRI sequences using deep convolutional neural network with unscented Kalman filter. Med Image Anal. (2021) 68:101916. 10.1016/j.media.2020.101916 33285484

[50] Ronneberger O Fischer P Brox T. U-net: convolutional networks for biomedical image segmentation. In: International Conference on Medical image computing and computer-assisted intervention. Munich: Springer (2015). p. 234–41.

[51] Salvador A Bellver M Campos V Baradad M Marques F Torres J. Recurrent neural networks for semantic instance segmentation. preprint arXiv:171200617. (2017). 31299493

[52] Xingjian S Chen Z Wang H Yeung DY Wong WK Woo Wc. Convolutional LSTM network: a machine learning approach for precipitation nowcasting. In: Advances in Neural Information Processing Systems. Montreal, QC: MIT Press (2015). p. 802–810.

[53] Isensee F Petersen J Klein A Zimmerer D Jaeger PF Kohl S. nnu-net: Self-adapting framework for u-net-based medical image segmentation. arXiv preprint arXiv:180910486. (2018).

[54] Chen Z Zhou H Xie X Lai J. Contour loss: Boundary-aware learning for salient object segmentation. arXiv preprint arXiv:190801975. (2019). 33201818 10.1109/TIP.2020.3037536

